# Genome-Wide Identification of Glutathione S-Transferase Family from *Dendrobium officinale* and the Functional Characterization of *DoGST5* in Cadmium Tolerance

**DOI:** 10.3390/ijms25158439

**Published:** 2024-08-02

**Authors:** Wu Jiang, Tao Wang, Man Zhang, Xiaojing Duan, Jiadong Chen, Yingying Liu, Zhengming Tao, Qiaosheng Guo

**Affiliations:** 1 Institute of Chinese Medicinal Materials, Nanjing Agricultural University, Nanjing 210095, China; jiangwu@zaas.ac.cn (W.J.); wt1344@njau.edu.cn (T.W.); 2019204055@njau.edu.cn (M.Z.); 2 Zhejiang Institute of Subtropical Crops, Zhejiang Academy of Agricultural Sciences, Wenzhou 325005, China; duanxj@zaas.ac.cn (X.D.); chenjd@zaas.ac.cn (J.C.); liuyy@zaas.ac.cn (Y.L.); yzszmtao@163.com (Z.T.)

**Keywords:** *Dendrobium officinale*, glutathione S-transferase, cadmium tolerance, reactive oxygen species

## Abstract

Glutathione S-transferases (GSTs) are members of a protein superfamily with diverse physiological functions, including cellular detoxification and protection against oxidative damage. However, there is limited research on GSTs responding to cadmium (Cd) stress. This study classified 46 GST genes in *Dendrobium officinale* (*D. officinale*) into nine groups using model construction and domain annotation. Evolutionary analysis revealed nine subfamilies with diverse physical and chemical properties. Prediction of subcellular localization revealed that half of the GST members were located in the cytoplasm. According to the expression analysis of GST family genes responding to Cd stress, *DoGST5* responded significantly to Cd stress. Transient expression of DoGST5-GFP in tobacco leaves revealed that DoGST5 was localized in the cytoplasm. *DoGST5* overexpression in Arabidopsis enhanced Cd tolerance by reducing Cd-induced H_2_O_2_ and O_2_^−^ levels. These findings demonstrate that *DoGST5* plays a critical role in enhancing Cd tolerance by balancing reactive oxygen species (ROS) levels, offering potential applications for improving plant adaptability to heavy metal stress.

## 1. Introduction

*Dendrobium officinale* (*D. officinale*), a renowned traditional Chinese medicinal herb, is widely distributed in subtropical regions [1]. It is considered the most precious species of the genus *Dendrobium* and is known for its diverse therapeutic effects [2]. In China, fresh *D. officinale* can be used as a high-quality dietary supplement and is processed into various products, such as *D. officinale* juice, functional wine, tea, capsules, and *D. officinale* powder, according to consumer preference. Currently, *D. officinale* cultivation spans over 6000 hectares nationwide, producing over 20,000 tons of fresh product annually [3,4]. However, *D. officinale* currently suffers from cadmium (Cd) toxicity. Field survey results indicate that the comprehensive pollution index of the heavy metal, Cd, in the cultivation substrate of *D. officinale* is 1.33, causing significant damage to the crop [5].

Glutathione S-transferases (GST) comprise a substantial gene family that encodes multifunctional enzymes primarily located in the cytoplasm. These enzymes play a significant role in oxidative stress metabolism by conjugating numerous substrates, including endobiotic and xenobiotic compounds, to detoxify harmful molecules [6,7]. The mechanism includes nucleophilic attack on nonpolar toxic compounds, facilitating their sequestration in vacuoles and eliminating toxic substances [8]. Investigation of the GST gene family has uncovered a substantial number of members in various plants, including *Arabidopsis* (57), poplar (81), rice (83), tomato (90), sweet potato (42), pumpkin (32), *Brassica rapa* (75), and *Physcomitrella patens* (37) [9,10,11,12,13,14,15,16,17]. This variation in and expansion of GST genes have been linked to duplication events [12,18,19]. Different classes of GSTs in plants have been identified based on their structural and functional characteristics. These classes include the tau, phi, theta, lambda, zeta, and gamma subunits of eukaryotic translation elongation factor 1B (EF1Bγ), dehydroascorbate reductase, and tetrachlorohydroquinone dehalogenase [9,12,15,20].

GST genes are involved in various physiological and developmental processes, as well as in the responses to biotic and abiotic stresses in numerous plant species [21,22,23,24,25]. The tau and phi classes have been extensively studied in the GST gene family. Overexpression of certain members of these two classes has been demonstrated to improve tolerance to drought, salt, oxidative stress, herbicides, heavy metals, and ultraviolet (UV) stressors in transgenic plants [26,27,28,29,30], indicating their protective role against abiotic stress. Increased transcript levels of tau class genes *NbGSTU1* and *NbGSTU3* were observed in *Nicotiana benthamiana* infected with *Colletotrichum* [31]. A recent study demonstrated that members of the tau class exhibit scavenging activity against highly toxic reactive carbonyl species (RCS), such as acrolein, and suggested their role in the defense system against RCS [32]. Genes from these two classes have also been implicated in the transport and metabolism of secondary compounds, such as Bz2 in maize [33], An9 in petunia [34], and TT19 in Arabidopsis [35]. Plants overexpressing GST genes, such as *Nt107* in tobacco and *LeGSTU2* in Arabidopsis, have demonstrated increased tolerance to various stresses [36,37].

Advancements in whole-genome sequencing and genome-wide analysis have enabled the identification and characterization of the GST gene family in various plant species. Research findings have revealed that GSTs, as a multigene family, have varying genes across different species. In *Arabidopsis*, 53 GST family members have been identified [38]. Seventy-nine members have been found in rice [12], 59 in *Gossypium raimondii*, 49 in *G. arboreum* [18], 90 in tomato [11], 54 in peach [39], 69 in apple [40], 97 in kiwifruit [41], and 57 in pear [42]. Although the GST gene family has been extensively characterized in various plant species, a comprehensive analysis of the GST family in *D. officinale* has not been reported. This study aimed to identify GST family members in *D. officinale* and analyze their expression patterns in response to Cd exposure. Subsequently, genes exhibiting significant responsiveness were selected for functional validation, laying the groundwork for further exploration of the mechanisms underlying Cd resistance mediated by DoGST family genes.

## 2. Results

### 2.1. Identification of the GST Protein Family in D. officinale

A total of 138 known GST protein sequences from *Arabidopsis* and rice (56 and 82 sequences, respectively) were obtained to construct a Hidden Markov Model (HMM) for a comprehensive understanding of the sequence characteristics of the GST family. Subsequently, the obtained sequences were processed using the Pfam A database and PfamScan software (version 1.6) for annotation, with the requirement that the selected sequences contain only two specific structures: GST_N and GST_C. Consequently, 46 members of the GST family were identified in *D. officinale* (Table S1).

### 2.2. Phylogenetic Analysis of the GST Gene Family

**Figure 1:**
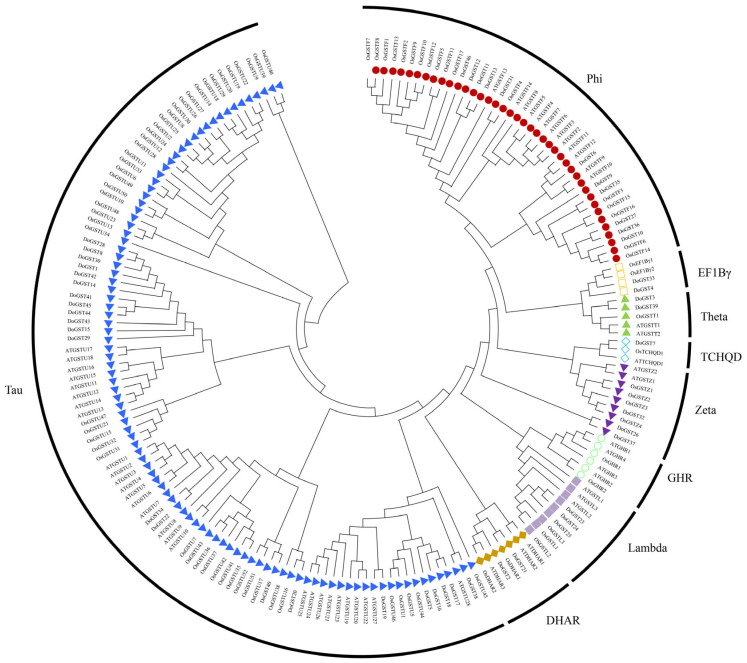
Phylogenetic tree of DoGSTs (contains GST family proteins of *Dendrobium officinale*, rice, and Arabidopsis).

Phylogenetic analysis of the GST gene family protein sequences from *D. officinale*, rice, and Arabidopsis was performed using the maximum likelihood method in MEGA software. After comparing the results, the GST proteins were categorized into nine groups, including phi, EF1Bγ, theta, TCHQD, zeta, GHR, lambda, DHAR, and tau. Among them, the tau branch comprised 22 family members, accounting for 47.8% and representing the largest branch of the GST gene family in *D. officinale*. The phi, EF1Bγ, theta, TCHQD, zeta, GHR, lambda, and DHAR branches contained 11, 2, 2, 1, 3, 0, 3, and 2 family members, respectively (Figure 1).

### 2.3. Physicochemical Property Analysis of the GST Gene Family

The ExPASy online platform was used to predict the physicochemical properties of the 46 members of the GST gene family in *D. officinale* to obtain data on molecular weight and other aspects (Table S2). The amino acid length ranged from 124 to 506, while the molecular weight ranged from 14,034.32 to 57,214.76 kilodaltons, and the isoelectric point ranged from 4.94 to 9.40. Eleven proteins, including DoGST2, DoGST27, DoGST35, DoGST45, DoGST19, DoGST26, DoGST24, DoGST37, DoGST3, DoGST12, and DoGST7, exhibited isoelectric points > 7, indicating alkaline proteins. However, the rest were acidic proteins with isoelectric points < 7. The instability index ranged from 20.01 to 55.94, with 20 proteins having indices > 40, indicating instability, and the remaining proteins having indices < 40, suggesting stability. The aliphatic index ranged from 57.06 to 111.71. The average hydrophilicity of the protein coefficient ranged from −1.216 to 0.156. DoGST9 and DoGST36 had coefficients of −1.216 and −0.563, respectively, indicating hydrophobic proteins. The remaining 44 proteins had coefficients > −0.5, indicating hydrophilic proteins (Table S2).

### 2.4. Analysis of Gene Structure and Conserved Protein Motifs in the GST Gene Family

**Figure 2:**
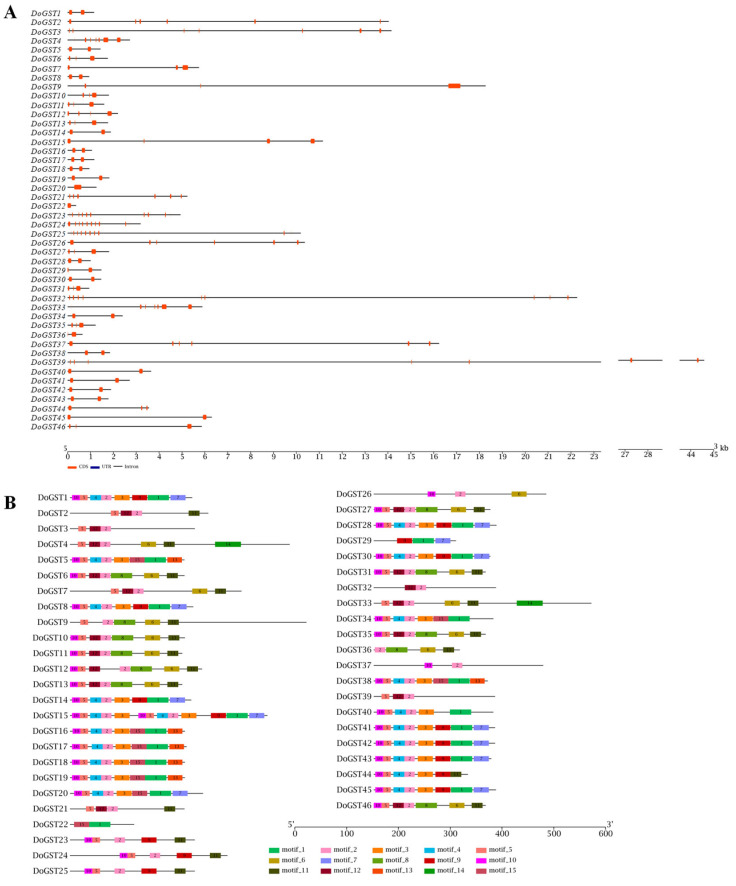
Gene structures and conserved motifs of DoGSTs. (**A**) Exon-intron organization of DoGST genes. Exons are shown as red rectangles; introns are shown as gray lines. (**B**) Composition and distributions of conserved motifs in DoGST proteins. The conserved motifs are represented by different numbers and rectangle colors.

Exons and introns play vital roles in understanding evolutionary relationships among species based on their positional information. The figure above depicts a comprehensive analysis of all of the identified genes. Genes generally contained 1–10 exons, with genes 23 and 25 having the largest number of exons, each containing 10 exons. Conversely, DoGST22 and DoGST36 had the lowest number of exons, each containing only one exon (Figure 2A). The MEME online tool for domain analysis results indicated that the GST family in *D. officinale* possessed 15 types of motifs. Each member contained at least two GST domains. Specifically, DoGST37 had the fewest motifs, containing only 2 motifs, while DoGST15 had the most, containing 13 motifs (Figure 2B).

### 2.5. Subcellular Localization Prediction of GST Proteins

Subcellular localization prediction was performed for the 46 members of the GST gene family in *D. officinale* using online tools. The results revealed that DoGST7 was predicted to localize to the plasma membrane. DoGST2, DoGST21, DoGST23, DoGST24, and DoGST25 were predicted to localize to the chloroplast. DoGST4, DoGST9, DoGST26, and DoGST37 were predicted to localize to the extracellular space, indicating secretion proteins. DoGST1, DoGST3, DoGST8, DoGST14, DoGST15, DoGST28, DoGST29, DoGST30, DoGST39, DoGST41, DoGST42, DoGST43, and DoGST45 were predicted to localize to the nucleus. The remaining 23 proteins were predicted to localize to the cytoplasm (Table S3).

### 2.6. Prediction of TF Binding Sites on the Promoters of the GST Gene Family

**Figure 3:**
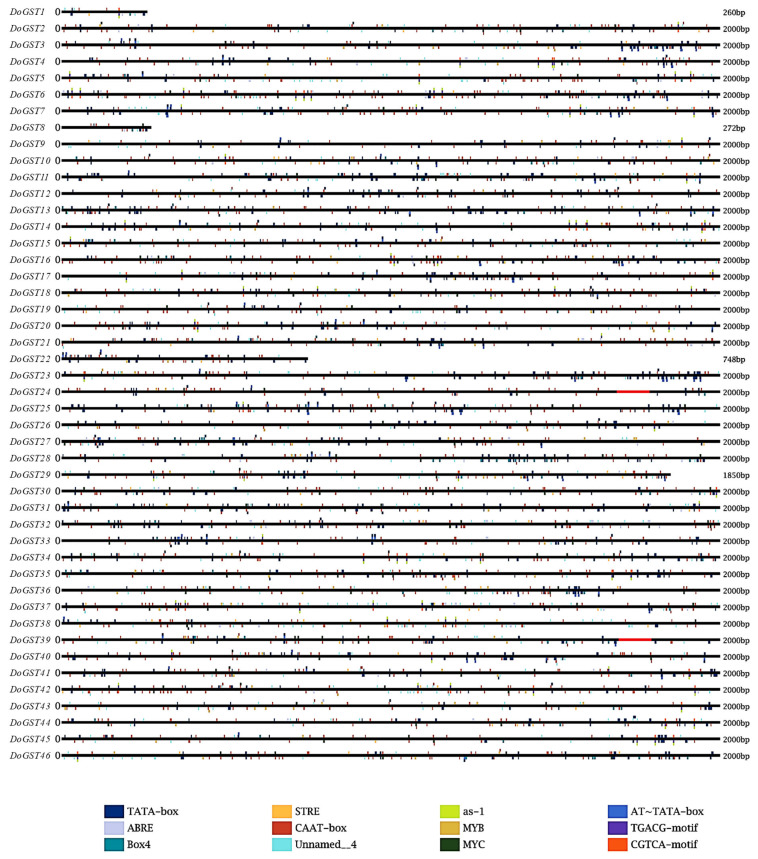
Prediction of TF binding sites on the promoter of GST family genes in *Dendrobium officinale.* The 2 kb upstream of the translation site was obtained for analysis, and the different colors represent the different of cis-acting elements in the promoter region of each *DoGST* gene.

The prediction results for the promoter elements revealed that the 46 members of the GST gene family in *D. officinale* contained a rich variety of *cis*-acting elements (Figure 3). These primarily included abiotic stress-related, hormone-responsive, and light-responsive elements. Notably, the promoters of *DoGST13* and *DoGST6* contained the highest number of *cis*-acting elements. These elements provided references for the future selection of upstream regulatory factor promoter fragments and interaction sites between regulatory factors and promoters (Figure 3).

### 2.7. Analysis of Expression Patterns of the GST Gene Family in Response to Cd

**Figure 4:**
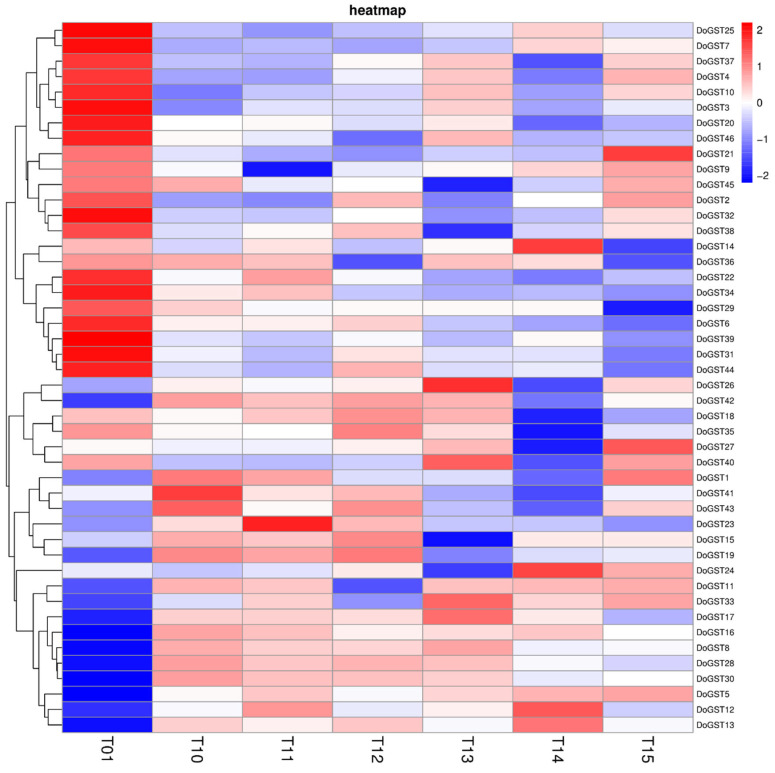
Expression analysis of GST family genes in response to Cd stress in *Dendrobium officinale*. T01 represents 0 mg·L^−1^ Cd treatment as the control. T10, T11, and T12 represent three biological repeats of 14 mg·L^−1^ Cd treatment for 15 days. T13, T14, and T15 represent three biological repeats of 14 mg·L^−1^ Cd treatment for 30 days.

The relative expression levels of GST genes in response to Cd stress were analyzed using transcriptome data from our previous research [43]. The results revealed that 23 GST family genes were generally downregulated in response to Cd, while the remaining 23 were upregulated (Figure 4). Furthermore, we found that the expression levels of *DoGST5*, *DoGST11*, and *DoGST33* increased with increasing Cd treatment time, while the expression levels of *DoGST15*, *DoGST18*, *DoGST19*, *DoGST23*, *DoGST28*, *DoGST30*, *DoGST41*, and *DoGST43* initially increased and then decreased after Cd treatment (Figure 4). This suggested that these genes may be involved in the Cd tolerance response of *D. officinale* and may be candidate genes for further analysis of Cd tolerance mechanisms.

### 2.8. Subcellular Localization Analysis of DoGST5 Protein

**Figure 5:**
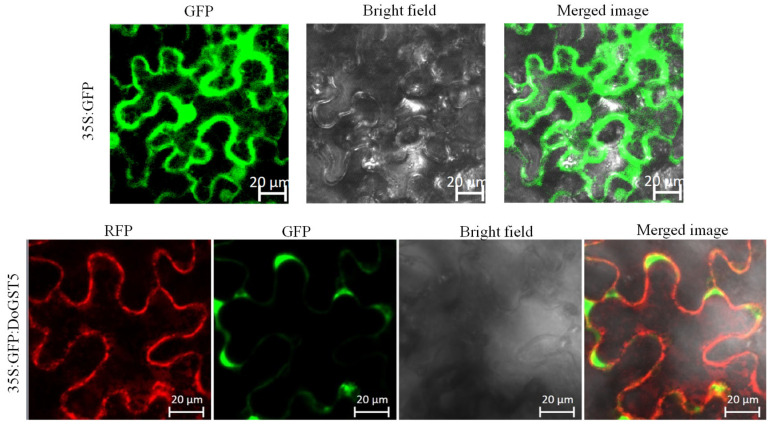
Subcellular localization of DoGST5 in *N. benthamiana* epidermal cells. Upper panel: 35S:GFP plasmid was transformed into *N. benthamiana* leaves. Lower panel: the fusion protein DoGST5::sGFP was transiently co-expressed with the RFP-rk plasma membrane marker into *N. benthamiana* leaves, bar = 20 μm.

Based on the expression pattern analysis, we selected the *D. officinale* GST family gene *DoGST5*, which showed a significant response to Cd, for further analysis. Firstly, the subcellular localization of this gene was analyzed. In this study, a fusion protein expression vector of DoGST5 protein fused with GFP protein (p35S::DoGST5::GFP) was constructed. Then, the recombinant plasmid was transferred into tobacco epidermal cells via Agrobacterium-mediated transformation and observed using confocal laser scanning microscopy. The results indicated that DoGST5 protein was expressed in the cytoplasm of tobacco epidermal cells, indicating that DoGST5 was localized in the cytoplasm (Figure 5). This was consistent with the predicted subcellular localization of DoGST5 protein.

### 2.9. Functional Analysis of DoGST5 in Cd Tolerance

**Figure 6:**
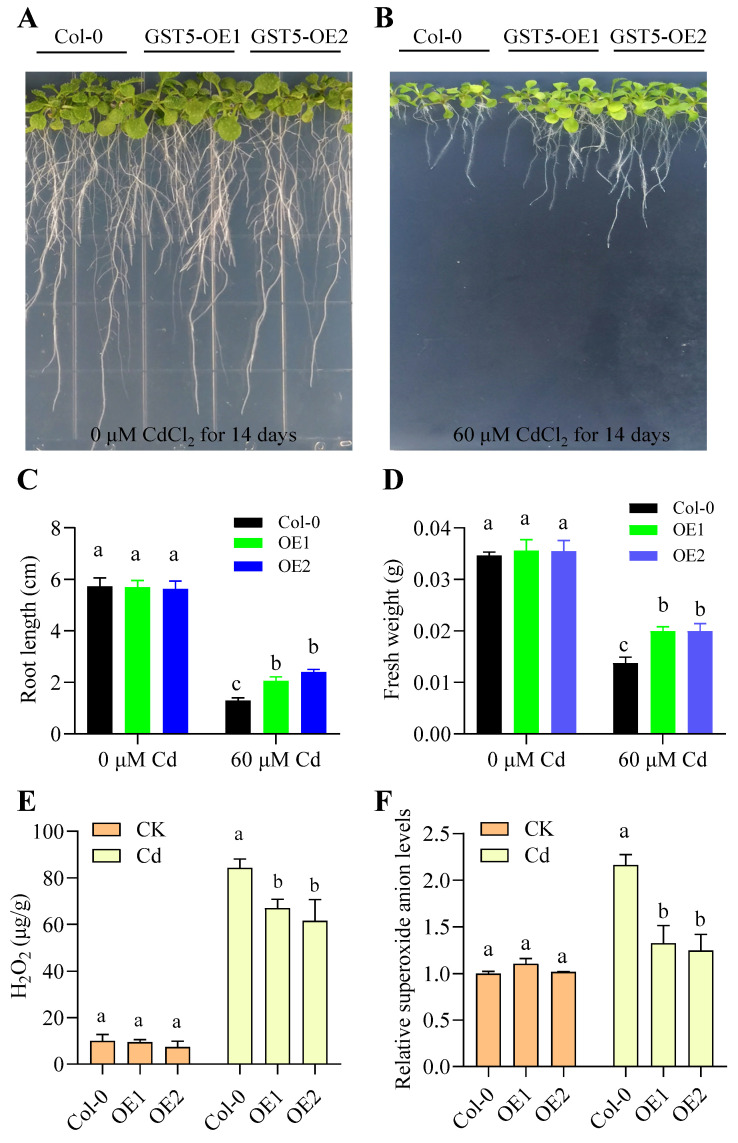
Overexpression of *DoGST5* enhances Cd tolerance in Arabidopsis by reducing the excessive accumulation of ROS induced by Cd stress. (**A**,**B**) Phenotypic analysis of Cd tolerance in Arabidopsis overexpressing *DoGST5*. Col-0 and *DoGST5* overexpression Arabidopsis lines were cultured on 0 and 60 μM 1/2 MS solid medium for 14 days. (**C**,**D**) Primary root length and fresh weight of Col-0 and *DoGST5* overexpression lines. Data are means ± SD (*n* = 5). (**E**,**F**) Overexpression of *DoGST5* in Arabidopsis reduces excessive ROS levels induced by Cd stress. Columns with different letters indicate significant differences (*p*  <  0.05) using one-way ANOVA with Tukey’s test for multiple comparisons.

Two transgenic Arabidopsis lines overexpressing *DoGST5* were identified using semi-quantitative PCR analysis. The results indicated that, at nearly identical brightness of the *AtActin* band, Col-0 did not exhibit amplification bands, while the two overexpression lines displayed brighter bands (Figure S1), indicating successful expression of *DoGST5* in both transgenic lines, making them suitable for subsequent experiments. Seeds of wild-type and transgenic homozygous Arabidopsis lines were sown on 1/2 MS solid culture medium containing 0 and 60 μM CdCl_2_ and placed in a growth chamber for two weeks. Phenotypic differences were observed, photographs were taken, and the root length and biomass were measured. As shown in Figure 6A,B, on culture medium containing 60 μM CdCl_2_, the *Arabidopsis* lines overexpressing *DoGST5* exhibited stronger resistance than the wild type. The root length of the overexpression lines was significantly longer than that of the wild type. The fresh weight of the overexpression lines was also significantly greater than that of the wild type. However, there were no significant differences in root length and biomass under control conditions (Figure 6C,D).

### 2.10. Enhancement of Plant Cd Tolerance by DoGST5 via Balancing ROS Levels

During GST reduction, GSH acts as an electron donor, reducing the organic peroxides in nucleotides and fatty acids to yield monohydric compounds. This reaction reduces the proportion of peroxides, effectively protects the cells, and prevents oxidative damage. Therefore, we measured the ROS content in wild-type and *DoGST5* overexpression lines of Arabidopsis. The results indicated that the H_2_O_2_ and O_2_^−^ levels in both *Arabidopsis* lines were increased significantly compared to the control group after Cd treatment. However, the *DoGST5* overexpression lines exhibited significantly lower H_2_O_2_ and O_2_^−^ levels than wild-type *Arabidopsis* after Cd treatment (Figure 6E,F), indicating that overexpression of *DoGST5* could reduce the excessive accumulation of ROS induced by Cd stress in plants, thereby reducing cell damage and enhancing plant Cd tolerance.

## 3. Discussion

Plant GSTs are multifunctional small soluble proteins widely distributed in organisms. Plant GSTs catalyze the reaction of the tripeptide, glutathione, transferring it to a common substrate, thus producing polar S-glutathione reaction products. Subsequently, these metabolites are transported to vacuoles, exerting detoxification effects. In this study, 46 members of the *D. officinale* GST gene family were identified based on the published genome and clustered into eight major classes, including phi, EF1Bγ, theta, TCHQD, zeta, lambda, DHAR, and tau. The number of GST genes in *D. officinale* was lower than that in *Arabidopsis* (66 GST genes) [44], rice (59 GST genes) [12], poplar (81 GST genes) [15], tomato (90 GST genes) [11], and soybean (101 GST genes) [45], but higher than that in potato (42 GST genes) [10]. The GST family members in *Arabidopsis* were divided into eight subfamilies, including phi, tau, theta, zeta, lambda, EF1Bγ, DHAR, and TCHQD [17]. *D. officinale* contains many GST subfamilies, suggesting that the *D. officinale* GST family may have undergone more sequence and functional differentiation during evolution. Phylogenetic studies have demonstrated that plant GSTs evolve gradually after plant differentiation, with the tau and phi subfamilies being particularly special and widely distributed in plants [46]. Tau and phi class genes play important roles in responses to salt, oxidative stress, herbicides, heavy metals, and UV stressors in plants [26,27,28,29,30]. Moreover, we found that *D. officinale* contains 12 and 14 genes for the tau and phi classes, respectively, belonging to the largest branches, indicating their crucial roles in stress response.

A study of *cis*-acting elements in the GST gene promoter regions revealed that many family members include MYC elements. External abiotic stress response regulation methods primarily include two types: ABA-dependent and non-dependent types. The roles of DRE and ABRE elements are significant in these two regulatory mechanisms. The first type primarily includes MYC and MYB elements, and the signaling system uses ABA to regulate MYC and other transcription factors, thereby expressing downstream stress-resistance genes [47]. For the second type, when the signal activates the sensor protein in the regulation process, it is transferred to DREB, which then binds to DRE to achieve expression [48]. ABRE is also important for plant abiotic stress. The induction of many specific proteins through ABA response mechanisms plays a regulatory role in plants. Consequently, these *cis*-acting elements may play important roles in the abiotic stress response of *D. officinale*.

GSTs are involved in multiple biochemical reactions and play a role in plant stress tolerance. Plant GSTs regulate reversible S-glutathionylation of protein thiol residues, protecting proteins from oxidation under stress [49]. Transgenic overexpression of GSTs has provided insight into their role in abiotic stress acclimatization. Overexpression of tau class GSTs from species like *Glycine soja*, *Arabidopsis*, and *Prosopis juliflora* resulted in enhanced stress tolerance through oxidative stress tolerance, induction of antioxidant machinery, and changes in redox state [36,50,51,52]. Overexpression of rice *OsGSTU4* led to pleiotropic effects, including reduced sensitivity to ABA and auxin, and upregulation of defense-related pathways [29]. Zeta class GSTs have also contributed to abiotic stress tolerance despite lacking significant GST/GPOX activity. Overexpression of *OsGSTZ2* in rice and ThGSTZ1 in *Tamarix hispida* increased stress tolerance and GST/GPOX activity [53,54,55,56]. GSTZ isomerase activity may reduce oxidant accumulation, aiding antioxidant machinery [57].

However, there are no reports on the resistance of the GST gene family in *D. officinale*. This study is the first time to report expression analysis of the GST gene family in *D. officinale* under Cd stress. Differentially expressed genes can serve as genetic resources for breeding *D. officinale* with heavy metal tolerance. The Cd-induced significant up-regulation of the *DoGST5* gene was preliminarily validated for Cd tolerance. Our evolutionary analysis showed that DoGST5 belongs to the tau family. Overexpression of *DoGST5* in *Arabidopsis* increased its tolerance to Cd by reducing levels of Cd-induced H_2_O_2_ and O_2_^−^. The results of the subcellular localization analysis showed that DoGST5 was localized in the cytoplasm. Its presence in the cytoplasm could be associated with detoxifying ROS and other harmful compounds, which is crucial for maintaining cellular homeostasis. The results indicated that overexpression of *DoGST5* can reduce excessive accumulation of ROS induced by Cd stress in plants, thereby alleviating plant cell damage and enhancing plant Cd tolerance. The studies mentioned above suggested that the protective functions of GSTs against abiotic stress are linked to protein abundance and catalytic activity. However, silencing *AtGSTU17* resulted in increased drought and salt stress tolerance, accompanied by anatomical and physiological changes such as a smaller stomatal aperture, lower transpiration rate, increased root growth, and higher ABA and GSH contents [58]. These findings highlight the signaling functions of GSTs beyond their catalytic activities, offering potential for enhancing plant robustness under abiotic stress.

## 4. Materials and Methods

### 4.1. Identification of Family Member Proteins

Based on the GST protein family sequences, a Hidden Markov Model (HMM) was constructed using HMMER software (version 3.0) to search for and identify the complete coding protein sequences in *D. officinale* and to comprehensively understand the family. For the GST family reference sequences, a BLASTp search was conducted against all protein sequences, with an e-value threshold set to 1 × 10^−10^. Sequences that successfully matched were considered candidate sequences, combined to obtain the candidate GST family protein sequences. The obtained sequences were subjected to structural domain annotation using PfamScan software (version 1.6) and the Pfam A database to confirm sequences containing only the GST_N and GST_C domains as the final GST sequences.

### 4.2. Evolutionary Tree Analysis of GSTs

After identification, a series of GST protein family sequences from other plants was obtained, enabling the construction of a maximum likelihood tree. The construction method involved multiple sequence alignment using MAFFT and subsequent tree building using MEGA software. To assess the confidence of the calculated evolutionary tree branches, the parameters were set as follows: substitution model—the Jones–Taylor–Thornton (JTT) model, site coverage cutoff—95%, bootstrap—1000 replicates. The other parameters were set to default.

### 4.3. Motif Structure Analysis of GST Family Proteins

MEME software (http://meme.nbcr.net/meme, version 4.11.2, accessed on 20 April 2020) was employed to analyze the conserved motifs in the GST family. The parameter for motif discovery was set as 15.

### 4.4. Gene Structure Analysis of GST Family

The obtained GST protein sequences were submitted to the MEME online tool (https://meme-suite.org/meme/tools/meme, accessed on 20 April 2020) to identify conserved motifs (Motifs), with the maximum value for motif parameters set to 10. The identified motifs from the protein sequences and structural information such as exons, introns, and untranslated regions (UTRs) extracted from the genome annotation files were visualized using the “Visualize Gene Structure” tool in TBtools software (version 0.6, accessed on 20 April 2020).

### 4.5. Subcellular Localization Prediction of GST Family

The subcellular localization of GST family members in *D. officinale* was predicted using ProtComp online software (http://www.softberry.com/berry.phtml?topic=protcomppl&group=help&subgroup=proloc, version 9.0, accessed on 20 April 2020).

### 4.6. Subcellular Localization

The CDS of *DoGST5* without a stop codon was cloned into the binary vector 35S::GFP (modified from pCAMBIA1300). The primers used are listed in Table S3. The resultant DoGST5-GFP vector was transformed into *Agrobacterium tumefaciens* and cultured in 4 mL of LB medium containing kanamycin resistance. The culture was shaken at 28 °C and 200 rpm until the OD_600_ reached approximately 0.6. After centrifugation at 8000 rpm for 6 min, the supernatant was discarded, and the same volume of MES solution (10 mM MES with pH = 5.6, 10 mM MgCl_2_, and 0.2 mM acetosyringone) was used for resuspension. After centrifugation, the pellet was resuspended in an equal volume of MES solution and incubated at room temperature for 2 h. Tabacco (cultivated for 45 days) was injected into the backs of the leaves using a sterile syringe and incubated for three days. The GFP fluorescence of the leaves near the injection site was observed using a laser confocal microscope (LSM880: Karl Zeiss, Leica, Germany).

### 4.7. Arabidopsis Transgenic Experiment

The CDS of *DoGST5* with a stop codon was cloned into the binary vector 35S::GFP. The primers used are listed in Table S3. The Agrobacterium carrying the *35S::DoGST5* plasmid was cultured in LB liquid medium (containing 50 μg·mL^−1^ kanamycin and 50 μg·mL^−1^ rifampicin) until the OD_600_ reached approximately 0.8. The cells were centrifuged and resuspended in an inoculation solution (1/2 MS liquid medium, 5% sucrose, 0.03% Silwet L-77 (Solarbio Science & Technology, Beijing, China), 0.01 μg·mL^−1^ 6-BA, and 20 mg·L^−1^ AS, pH adjusted to 5.7 with KOH). The plants were then immersed in the suspension solution for 5 min. After 24 h of dark treatment, the plants were transferred to normal growth conditions. Positive seedlings were screened on 1/2 MS solid medium containing 50 μg·mL^−1^ hygromycin. T3 generation seeds were obtained for subsequent experiments.

### 4.8. Determination of H_2_O_2_ and O_2_^−^ Concentrations

The H_2_O_2_ and O_2_^−^ concentrations were measured using assay kits (Suzhou Keming Biotech Co. Ltd., Jiangsu, China). For the H_2_O_2_ assay, 0.1 g of leaves was added to 1 mL of reagent 1, homogenized in an ice bath, then transferred to an EP tube, adjusted to 1 mL with reagent 1, and centrifuged at 8000× *g* for 10 min at 4 °C. For the O_2_^−^ assay, 100 mg of frozen leaves was added to 1 mL of extracting solution, homogenized in an ice bath, and centrifuged at 10,000× *g* for 20 min at 4 °C. The absorbance of the supernatant was measured at 415 nm for H_2_O_2_ and 530 nm for O_2_^−^.

## 5. Conclusions

In conclusion, this study investigated the GST gene family in *D. officinale*, particularly its response to Cd stress. We identified 46 GST genes and classified them into nine groups, with the tau group being the most prominent. Phylogenetic and physicochemical analyses revealed diverse properties of these genes. Subcellular localization predictions indicated that half of the GSTs were cytoplasmic. Expression analysis under Cd stress identified *DoGST5* as significantly responsive. The transient expression of DoGST5-GFP in tobacco confirmed its cytoplasmic localization. Overexpression of *DoGST5* in *Arabidopsis* enhanced Cd tolerance by reducing ROS levels and mitigating cell damage. Functional analyses revealed that *DoGST5* overexpression improved root length and biomass under Cd stress, while ROS measurements exposed lower H_2_O_2_ and O_2_^−^ levels in transgenic lines than in wild-type plants. These findings suggest that *DoGST5* plays a vital role in enhancing Cd tolerance by balancing ROS levels, offering potential applications for improving plant resilience to heavy metal stress.

## Data Availability

Data are contained within the article and Supplementary Materials.

## Supplementary Materials

The following supporting information can be downloaded at: https://www.mdpi.com/article/10.3390/ijms25158439/s1.

## References

[1] Ng T.B., Liu J., Wong J.H., Ye X., Wing Sze S.C., Tong Y., Zhang K.Y. (2019). Review of research on Dendrobium, a prized folk medicine. Appl. Microbiol. Biotechnol..

[2] Bulpitt C.J., Li Y., Bulpitt P.F., Wang J. (2007). The use of orchids in Chinese medicine. J. R. Soc. Med..

[3] Zhu B., Wu L., Wan H., Yang K., Si J., Qin L. (2018). Fungal elicitors stimulate biomass and active ingredients accumulation in *Dendrobium catenatum* plantlets. Biologia.

[4] Yu Z., Liao Y., Teixeira da Silva J.A., Yang Z., Duan J. (2018). Differential accumulation of anthocyanins in *Dendrobium officinale* stems with red and green peels. Int. J. Mol. Sci..

[5] Xu L., Zheng W., Wang X., Song X., Wu W., Wu C. (2015). Monitoring and evaluation for heavy metals of *Dendrobium officinale* Kimura et Migo and its origin environment. Acta Agric. Zhejiangensis.

[6] Edwards R., Dixon D.P. (2005). Plant glutathione transferases. Method. Enzymol..

[7] Hayes J.D., Flanagan J.U., Jowsey I.R. (2005). Glutathione transferases. Annu. Rev. Pharmcol. Toxicol..

[8] Coleman J., Blake-Kalff M., Davies E. (1997). Detoxification of xenobiotics by plants: Chemical modification and vacuolar compartmentation. Trends Plant Sci..

[9] Dixon D.P., Edwards R. (2010). Roles for stress-inducible lambda glutathione transferases in flavonoid metabolism in plants as identified by ligand fishing. J. Biol. Chem..

[10] Ding N., Wang A., Zhang X., Wu Y., Wang R., Cui H., Huang R., Luo Y. (2017). Identification and analysis of glutathione S-transferase gene family in sweet potato reveal divergent GST-mediated networks in aboveground and underground tissues in response to abiotic stresses. BMC Plant Biol..

[11] Islam S., Rahman I.A., Islam T., Ghosh A. (2017). Genome-wide identification and expression analysis of glutathione S-transferase gene family in tomato: Gaining an insight to their physiological and stress-specific roles. PLoS ONE.

[12] Jain M., Ghanashyam C., Bhattacharjee A. (2010). Comprehensive expression analysis suggests overlapping and specific roles of rice glutathione S-transferase genes during development and stress responses. BMC Genom..

[13] Abdul Kayum M., Nath U.K., Park J.I., Biswas M.K., Choi E.K., Song J.Y., Kim H.T., Nou I.S. (2018). Genome-wide identification, characterization, and expression profiling of glutathione S-transferase (GST) family in pumpkin reveals likely role in cold-stress tolerance. Genes.

[14] Khan N., Hu C.M., Amjad Khan W., Hou X. (2018). Genome-wide identification, classification, and expression divergence of glutathione-transferase family in *Brassica rapa* under multiple hormone treatments. Biomed. Res. Int..

[15] Lan T., Yang Z.L., Yang X., Liu Y.J., Wang X.R., Zeng Q.Y. (2009). Extensive functional diversification of the populus glutathione S-transferase supergene family. Plant Cell.

[16] Liu Y.J., Han X.M., Ren L.L., Yang H.L., Zeng Q.Y. (2013). Functional divergence of the glutathione S-transferase supergene family in *Physcomitrella patens* reveals complex patterns of large gene family evolution in land plants. Plant Physiol..

[17] Sappl P.G., Carroll A.J., Clifton R., Lister R., Whelan J., Harvey Millar A., Singh K.B. (2009). The Arabidopsis glutathione transferase gene family displays complex stress regulation and co-silencing multiple genes results in altered metabolic sensitivity to oxidative stress. Plant J..

[18] Dong Y., Li C., Zhang Y., He Q., Daud M.K., Chen J., Zhu S. (2016). Glutathione s-transferase gene family in *Gossypium raimondii* and *G. arboreum*: Comparative genomic study and their expression under salt stress. Front. Plant Sci..

[19] Wang L., Qian M., Wang R., Wang L., Zhang S. (2018). Characterization of the glutathione S-transferase (GST) gene family in *Pyrus bretschneideri* and their expression pattern upon superficial scald development. Plant Growth Regul..

[20] Oakley A.J. (2005). Glutathione transferases: New functions. Curr. Opin. Struct. Biol..

[21] Nianiou-Obeidat I., Madesis P., Kissoudis C., Voulgari G., Chronopoulou E., Tsaftaris A., Labrou N.E. (2017). Plant glutathione transferase-mediated stress tolerance: Functions and biotechnological applications. Plant Cell Rep..

[22] Du J., Ren J., Ye X., Hou A., Fu W., Mei F., Liu Z. (2018). Genome-wide identification and expression analysis of the glutathione S-transferase (GST) family under different developmental tissues and abiotic stresses in Chinese cabbage *(Brassica rapa* ssp. pekinensis). Peer J. Preprints.

[23] Gullner G., Komives T., Király L., Schröder P. (2018). Glutathione S-transferase enzymes in plant-pathogen interactions. Front. Plant Sci..

[24] Kumar S., Trivedi P.K. (2018). Glutathione S-transferases: Role in combating abiotic stresses including arsenic detoxification in plants. Front. Plant Sci..

[25] Wahibah N.N., Tsutsui T., Tamaoki D., Sato K., Nishiuchi T. (2018). Expression of barley *glutathione s-transferase13* gene reduces accumulation of reactive oxygen species by trichothecenes and paraquat in arabidopsis plants. Plant Biotechnol..

[26] Karavangeli M., Labrou N.E., Clonis Y.D., Tsaftaris A. (2005). Development of transgenic tobacco plants overexpressing maize glutathione S-transferase I for chloroacetanilide herbicides phytoremediation. Biomol. Eng..

[27] Benekos K., Kissoudis C., Nianiou-Obeidat I., Labrou N., Madesis P., Kalamaki M., Makris A., Tsaftaris A. (2010). Overexpression of a specific soybean GmGSTU4 isoenzyme improves diphenyl ether and chloroacetanilide herbicide tolerance of transgenic tobacco plants. J. Biotechnol..

[28] Jha B., Sharma A., Mishra A. (2011). Expression of *SbGSTU* (tau class glutathione S-transferase) gene isolated from *Salicornia brachiata* in tobacco for salt tolerance. Mol. Biol. Rep..

[29] Sharma R., Sahoo A., Devendran R., Jain M. (2014). Over-expression of a rice tau class glutathione S-transferase gene improves tolerance to salinity and oxidative stresses in Arabidopsis. PLoS ONE.

[30] Srivastava D., Verma G., Chauhan A.S., Pande V., Chakrabarty D. (2019). Rice (*Oryza sativa* L.) tau class glutathione S-transferase (OsGSTU30) overexpression in Arabidopsis thaliana modulates a regulatory network leading to heavy metal and drought stress tolerance. Metallomics.

[31] Dean J.D., Goodwin P.H., Hsiang T. (2005). Induction of glutathione S-transferase genes of *Nicotiana benthamiana* following infection by *Colletotrichum destructivum* and *C. orbiculare* and involvement of one in resistance. J. Exp. Bot..

[32] Mano J.I., Kanameda S., Kuramitsu R., Matsuura N., Yamauchi Y. (2019). Detoxification of reactive carbonyl species by glutathione transferase tau isozymes. Front. Plant Sci..

[33] Marrs K.A., Alfenito M.R., Lloyd A.M., Walbot V. (1995). A glutathione S-transferase involved in vacuolar transfer encoded by the maize gene *Bronze-2*. Nature.

[34] Mueller L.A., Goodman C.D., Silady R.A., Walbot V. (2000). AN9, a Petunia Glutathione S-Transferase Required for Anthocyanin Sequestration, Is a Flavonoid-Binding Protein. Plant Physiol..

[35] Sun Y., Li H., Huang J.R. (2012). Arabidopsis TT19 functions as a carrier to transport anthocyanin from the cytosol to tonoplasts. Mol. Plant.

[36] Roxas V.P., Lodhi S.A., Garrett D.K., Mahan J.R., Allen R.D. (2000). Stress tolerance in transgenic tobacco seedlings that overexpress glutathione S-transferase/glutathione peroxidase. Plant Cell Physiol..

[37] Xu J., Xing X.J., Tian Y.S., Peng R.H., Xue Y., Zhao W., Yao Q.H. (2015). Transgenic Arabidopsis plants expressing tomato glutathione S-transferase showed enhanced resistance to salt and drought stress. PLoS ONE.

[38] Sappl P.G., Oñate-Sánchez L., Singh K.B., Millar A.H. (2004). Proteomic analysis of glutathione S-transferases of *Arabidopsis thaliana* reveals differential salicylic acid-induced expression of the plant-specific phi and tau classes. Plant Mol. Biol..

[39] Zhao Y., Dong W., Wang K., Zhang B., Allan A.C., Lin-Wang K., Chen K., Xu C. (2017). Differential sensitivity of fruit pigmentation to ultraviolet light between two peach cultivars. Front. Plant Sci..

[40] Jiang S., Chen M., He N., Chen X., Wang N., Sun Q., Zhang T., Xu H., Fang H., Wang Y. (2019). *MdGSTF6*, activated by MdMYB1, plays an essential role in anthocyanin accumulation in apple. Hortic. Res..

[41] Liu Y., Qi Y., Zhang A., Wu H., Liu Z., Ren X. (2019). Molecular cloning and functional characterization of AcGST1, an anthocyanin-related glutathione S-transferase gene in kiwifruit (*Actinidia chinensis*). Plant. Mol. Biol..

[42] Li B., Zhang X., Duan R., Han C., Yang J., Wang L., Wang S., Su Y., Wang L., Dong Y. (2022). Genomic analysis of the glutathione s-transferase family in Pear (*Pyrus communis*) and functional identification of *PcGST57* in anthocyanin accumulation. Int. J. Mol. Sci..

[43] Jiang W., Wu Z., Wang T., Mantri N., Huang H., Li H., Tao Z., Guo Q. (2020). Physiological and transcriptomic analyses of cadmium stress response in Dendrobium officinale seedling. Plant Physiol. Bioch..

[44] Dixon D.P., Edwards R. (2010). Glutathione transferases. Arab. Book.

[45] Liu H.J., Tang Z.X., Han X.M., Yang Z.L., Zhang F.M., Yang H.L., Liu Y.J., Zeng Q.Y. (2015). Divergence in enzymatic activities in the soybean GST supergene family provides new insight into the evolutionary dynamics of whole-genome duplicates. Mol. Biol. Evol..

[46] Frova C. (2003). The plant glutathione transferase gene family: Genomic structure, functions, expression and evolution. Physiol. Plant..

[47] Agarwal M., Hao Y., Kapoor A., Dong C.H., Fujii H., Zheng X., Zhu J.K. (2006). A R2R3 type MYB transcription factor is involved in the cold regulation of CBF genes and in acquired freezing tolerance. J. Biol. Chem..

[48] Shinozaki K., Yamaguchi-Shinozaki K. (2000). Molecular responses to dehydration and low temperature: Differences and cross-talk between two stress signaling pathways. Curr. Opin. Plant Biol..

[49] Mieyal J.J., Chock P.B. (2012). Posttranslational modification of cysteine in redox signaling and oxidative stress: Focus on S-glutathionylation. Antioxid. Redox Sign..

[50] George S., Venkataraman G., Parida A. (2010). A chloroplast-localized and auxin-induced glutathione S-transferase from phreatophyte Prosopis juliflora confer drought tolerance on tobacco. J. Plant Physiol..

[51] Jia B., Sun M., Sun X., Li R., Wang Z., Wu J., Wei Z., DuanMu H., Xiao J., Zhu Y. (2016). Overexpression of *GsGSTU13* and *SCMRP* in *Medicago sativa* confers increased salt–alkaline tolerance and methionine content. Physiol. Plant.

[52] Xu J., Tian Y.S., Xing X.J., Peng R.H., Zhu B., Gao J.J., Yao Q.H. (2016). Over-expression of *AtGSTU19* provides tolerance to salt, drought and methyl viologen stresses in *Arabidopsis*. Physiol. Plant..

[53] Kim S.I., Andaya V.C., Tai T.H. (2011). Cold sensitivity in rice (*Oryza sativa* L.) is strongly correlated with a naturally occurring I99V mutation in the multifunctional glutathione transferase isoenzyme GSTZ2. Biochem. J..

[54] Takesawa T., Ito M., Kanzaki H., Kameya N., Nakamura I. (2022). Over-expression of ζ glutathione S-transferase in transgenic rice enhances germination and growth at low temperature. Mol. Breed..

[55] Yang G., Wang Y., Xia D., Gao C., Wang C., Yang C. (2014). Overexpression of a GST gene (*ThGSTZ1*) from *Tamarix hispida* improves drought and salinity tolerance by enhancing the ability to scavenge reactive oxygen species. Plant Cell Tiss. Org..

[56] Yang Q., Liu Y.J., Zeng Q.Y. (2014). Biochemical functions of the glutathione transferase supergene family of *Larix kaempferi*. Plant Physiol. Biochem..

[57] Blackburn A.C., Matthaei K.I., Lim C., Taylor M.C., Cappello J.Y., Hayes J.D., Anders M.W., Board P.G. (2006). Deficiency of glutathione transferase zeta causes oxidative stress and activation of antioxidant response pathways. Mol. Pharmacol..

[58] Chen J.H., Jiang H.W., Hsieh E.J., Chen H.Y., Chien C.T., Hsieh H.L., Lin T.P. (2012). Drought and salt stress tolerance of an Arabidopsis glutathione *S*-transferase U17 knockout mutant are attributed to the combined effect of glutathione and abscisic acid. Plant Physiol..

