# Supplementary figures and images for: Genome-Wide Identification of Glutathione S-Transferase Family from Dendrobium officinale and the Functional Characterization of DoGST5 in Cadmium Tolerance
Source: Int J Mol Sci. 2024 Aug 2;25(15):8439. doi: 10.3390/ijms25158439 (PMC11313178; doi:10.3390/ijms25158439)

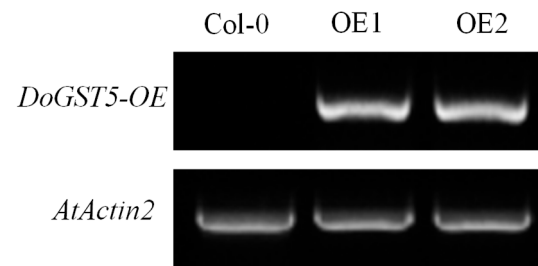

Figure S1. Semi-quantitative PCR to verify the expression of *DoGST5* in Arabidopsis.

Supplement: Supplementary file 1 [file ijms-25-08439-s001.zip › ijms-3126192-supplementary/Supplementary Files/Figure S1.pdf]
